# Supplementary material for: Does Influenza Vaccination during Pregnancy Have Effects on Non-Influenza Infectious Morbidity? A Systematic Review and Meta-Analysis of Randomised Controlled Trials
Source: Vaccines (Basel). 2021 Dec 8;9(12):1452. doi: 10.3390/vaccines9121452 (PMC8707251; doi:10.3390/vaccines9121452)
Supplement: Supplementary file 1 [file vaccines-09-01452-s001.zip › vaccines-1456503-supplementary.pdf]

Table S1. Available information on non-specific outcomes in the randomised controlled trials of influenza vaccination during pregnancy**.**

Country	Author, year	Maternal mortality	Maternal mortality from presumed infectious causes	Maternal non-influenza infectious adverse events	Miscarriages and stillbirths	Child mortality	Child mortality from presumed infectious causes	Child non-influenza infectious adverse events
**Bangladesh**	Zaman et al. 2008[1]	In appendix (Table 3, only one death due to an accident)	In appendix (Table 3, only one death due to an accident)	In appendix (Table 3, adverse events, hospitalisations)	In appendix (Table 3, adverse events, stillbirths)	In appendix (Table 3, adverse events. perinatal mortality)	In appendix (Table 3, adverse events perinatal mortality)	In appendix (Table 3, adverse events. hospitalisations)
**South Africa**	Madhi et al. 2014[2]	In appendix (S13+S18)	In appendix (S13+S18)	In appendix (S16+S22, hospitalisations)	In main tables	In appendix (S12+S19)	In appendix (S12+S19)	In appendix (S17+S23, hospitalisations)
**Mali**	Tapia et al. 2016[3]	In main text	In main text	In appendix (S6, serious adverse events))	In appendix (S7)	In appendix (S7)	In appendix (S8)	In appendix (S7, serious adverse events)
**Nepal**	Steinhoff et al. 2017[4]	In main text	In main text	N/A	In main text	In appendix (S2)	N/A	N/A

NA= not available.

Table S2. Risk of bias assessment.

Author, year	Random sequence generation	Allocation concealment	Blinding of participants and personnel	Blinding of outcome assessment	Incomplete outcome data	Selective reporting	Risk of bias
Zaman et al. 2008[1]							Low
Madhi et al. 2014[2]		?					Low
Tapia et al. 2016[3]				?			Low
Steinhoff et al. 2017[4]							Low

Figure S1: PRISMA flow diagram.

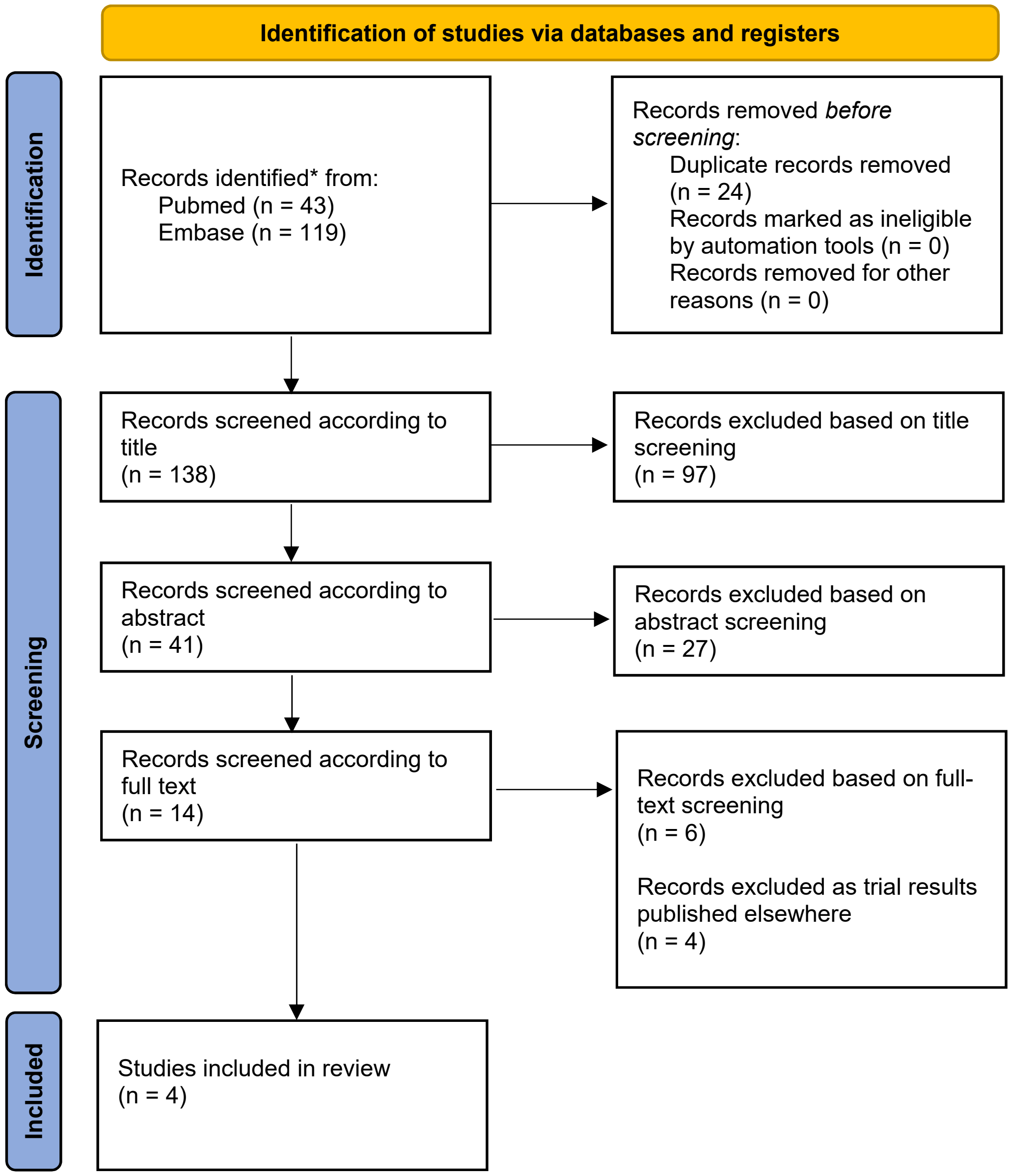

Adapted from: Page MJ, McKenzie JE, Bossuyt PM, Boutron I, Hoffmann TC, Mulrow CD, et al. The PRISMA 2020 statement: an updated guideline for reporting systematic reviews. BMJ 2021;372:n71. doi: 10.1136/bmj.n71

* We searched PubMed and Embase for RCTs published before 30 December 2020, using the search terms related to 'Influenza Vaccines' AND 'Pregnancy' AND ('Pregnancy Outcome' OR 'Immunity, Heterologous') AND 'Randomised Trial'.

Search string PubMed:

('Influenza Vaccines'[Mesh] OR 'influenza vaccination' OR 'influenza vaccine' OR 'influenza vaccines' OR 'influenza immunisation' OR 'influenza immunization' OR 'flu vaccination' OR 'flu vaccine' OR 'flu vaccines' OR 'flu immunisation' OR 'flu immunization')
OR
(('Immunity, Maternally-Acquired '[Mesh] OR 'maternal immunisation' OR 'maternal immunization' OR 'maternal vaccination' OR 'maternal vaccine')
AND
'Influenza')
AND

(‘Pregnancy’[Mesh] OR ‘Maternal Exposure’[Mesh] OR pregnancy OR pregnant OR gestation OR gravidity OR gravid)
AND
(‘Pregnancy Complications’[Mesh] OR ‘Pregnancy Outcome’[Mesh] OR ‘Infant’[Mesh] OR ‘Fetal Mortality’[Mesh] OR ‘Infant Mortality’[Mesh] OR ‘Mortality, Premature’[Mesh] OR ‘Child Mortality’[Mesh] OR ‘Infant Death’[Mesh] OR ‘Aborted Fetus’[Mesh] OR ‘Birth Weight’[Mesh] OR ‘Child Health’[Mesh] OR miscarriage OR ‘pregnancy loss’ OR abortion OR stillbirth OR death OR mortality OR ‘preterm birth’ OR ‘preterm infant’ OR ‘premature birth’ OR ‘premature infant’ OR ‘birth weight’ OR ‘pregnancy complications’ OR ‘pregnancy complication’ OR ‘pregnancy outcome’ OR ‘pregnancy outcomes’ OR ‘birth outcome’ OR ‘birth outcomes’ OR morbidity OR survival OR ‘side effect’ OR ‘side effects’ OR ‘adverse effect’ OR ‘adverse effects’ OR ‘adverse event’ OR ‘adverse events’ OR ‘non-specific effects’ OR ‘non-specific effect’ OR ‘Immunity, Heterologous’[Mesh] OR ‘Heterologous effects’ OR ‘Heterologous effect’ OR ‘off-target effect’ OR ‘off-target effects’ OR ‘heterologous immunity’ OR ‘non-targeted immunity’ OR ‘non targeted immunity’)
AND
(‘Randomised trial*’ OR ‘Randomized trial*’ OR ‘Randomised controlled trial*’ OR ‘Randomized controlled trial’)

Search string Embase:

('influenza vaccination'/exp OR 'influenza vaccine'/exp OR ’influenza vaccination’ OR ’influenza vaccine’ OR ’influenza vaccines’ OR ’influenza immunisation’ OR ’influenza immunization’ OR ’flu vaccination’ OR ’flu vaccine’ OR ’flu vaccines’ OR ’flu immunisation’ OR ’flu immunization’)
OR
(('maternal immunization'/exp OR 'maternal vaccination'/exp OR ‘maternal immunisation’ OR ‘maternal immunization’ OR ‘maternal vaccination’ OR ‘maternal vaccine’)
AND ‘Influenza’)
AND
('pregnancy'/exp OR ‘pregnant woman’/exp OR ‘prenatal exposure’/exp OR ‘prenatal drug exposure’/exp OR ‘maternal exposure’/exp OR pregnancy OR pregnant OR gestation OR gravidity OR gravid)
AND
('pregnancy disorder'/exp OR ‘parameters concerning the fetus, newborn and pregnancy’/exp OR 'infant'/exp OR ‘mortality’/exp OR ‘morbidity’/exp OR 'child death'/exp OR 'fetus death'/exp OR ‘embryo death’/exp OR ‘perinatal death’/exp OR ‘child health’/exp OR ‘adverse event’/exp OR ‘side effect’/exp OR miscarriage OR ’pregnancy loss’ OR abortion OR stillbirth OR death OR mortality OR ‘preterm birth’ OR ‘preterm infant’ OR ‘premature birth’ OR ‘premature infant’ OR ‘birth weight’ OR ‘pregnancy complications’ OR ’pregnancy complication’ OR ’pregnancy outcomes’ OR ‘pregnancy outcome’ OR ’birth outcome’ OR ’birth outcomes’ OR morbidity OR survival OR ’side effect’ OR ’side effects’ OR ’adverse effect’ OR ’adverse effects’ OR ‘adverse event’ OR ‘adverse events’ OR ‘non-specific effects’ OR ‘non-specific effect’ OR 'heterologous immunity'/exp OR ‘Heterologous effects’ OR ‘Heterologous effect’ OR ‘off-target effect’ OR ‘off-target effects’ OR ‘heterologous immunity’ OR ‘non-targeted immunity’)
AND
(‘Randomised trial*’ OR ‘Randomized trial*’ OR ‘Randomised controlled trial*’ OR ‘Randomized controlled trial*’)

Figure S2. The effect of influenza vaccination in pregnancy on miscarriages and stillbirths.

Study or Subgroup	Experimental Events	Experimental Total	Control Events	Control Total	Weight	Risk Ratio M-H, Fixed, 95% CI
Bangladesh	3	172	0	168	0.6%	6.84 [0.36, 131.38]
Mali	24	2108	30	2085	38.3%	0.79 [0.46, 1.35]
Nepal	38	1847	34	1846	43.2%	1.12 [0.71, 1.77]
South Africa, HIV neg	18	1062	14	1054	17.8%	1.28 [0.64, 2.55]
South Africa, HIV pos	0	100	0	94		Not estimable
**Total (95% CI)**		**5289**		**5247**	**100.0%**	**1.06 [0.78, 1.44]**
Total events	83		78			

Heterogeneity: Chi² = 3.00, df = 3 (P = 0.39); I² = 0%

Test for overall effect: Z = 0.36 (P = 0.72)

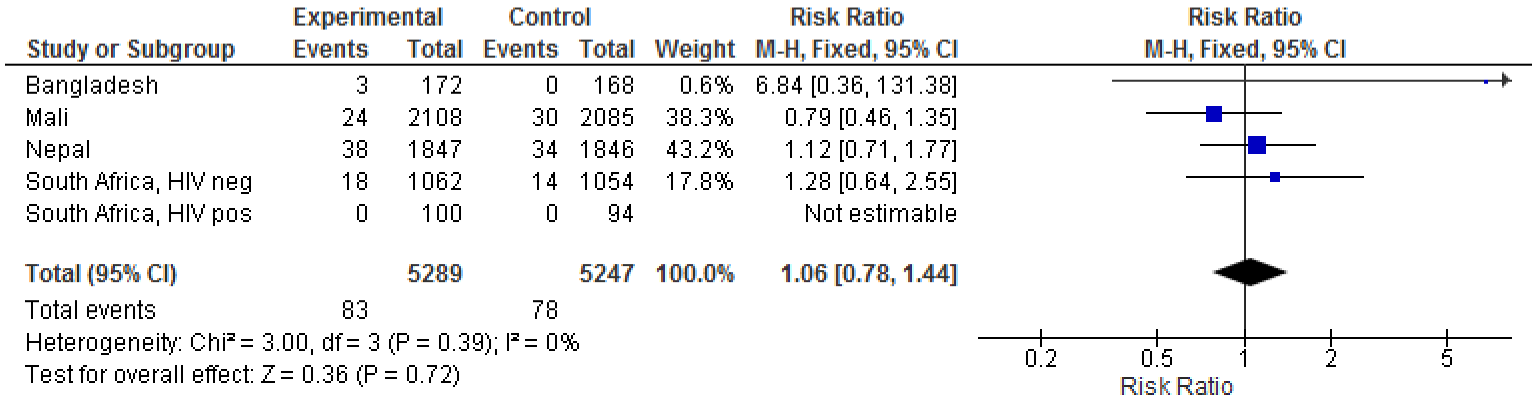

Figure S3. The effect of influenza vaccination in pregnancy on infant mortality.

Study or Subgroup	Experimental Events	Experimental Total	Control Events	Control Total	Weight	Risk Ratio M-H, Fixed, 95% CI
Bangladesh	1	172	2	168	1.7%	0.49 [0.04, 5.34]
Mali	52	2108	37	2085	31.1%	1.39 [0.92, 2.11]
Nepal	60	1847	51	1846	42.7%	1.18 [0.81, 1.70]
South Africa, HIV neg	15	1062	21	1054	17.6%	0.71 [0.37, 1.37]
South Africa, HIV pos	5	100	8	94	6.9%	0.59 [0.20, 1.73]
**Total (95% CI)**		**5289**		**5247**	**100.0%**	**1.11 [0.87, 1.41]**
Total events	133		119			
Heterogeneity: Chi² = 4.78, df = 4 (P = 0.31); I² = 16%						
Test for overall effect: Z = 0.82 (P = 0.41)						

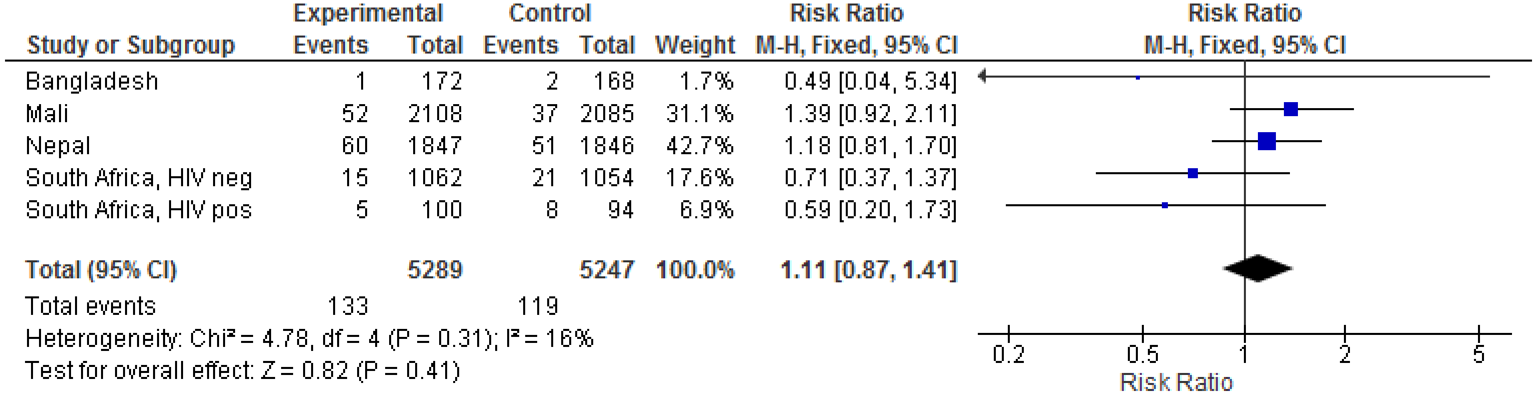

*Excluding miscarriages and stillbirths

Notes: In Nepal, there was inconsistent reporting of infant deaths in the abstract (61 IIV, 50 control), trial profile, and appendix (both 60 IIV, 51 control). For the present study, we used 60 vs. 51.

Figure S4. The effect of influenza vaccination in pregnancy on infant mortality from possible infection-related causes.

	Experimental		Control			Risk Ratio
Study or Subgroup	Events	Total	Events	Total	Weight	M-H, Fixed, 95% CI
Bangladesh	0	172	0	168		Not estimable
Mali	17	2108	14	2085	60.7%	1.20 [0.59, 2.43]
South Africa, HIV neg	3	1062	6	1054	26.0%	0.50 [0.12, 1.98]
South Africa, HIV pos	2	100	3	94	13.3%	0.63 [0.11, 3.67]
**Total (95% CI)**		**3442**		**3401**	**100.0%**	**0.94 [0.53, 1.68]**
Total events	22		23			

Heterogeneity: $Chi^2 = 1.49$, $df = 2$ ($P = 0.48$); $I^2 = 0\%$
Test for overall effect: $Z = 0.20$ ($P = 0.84$)

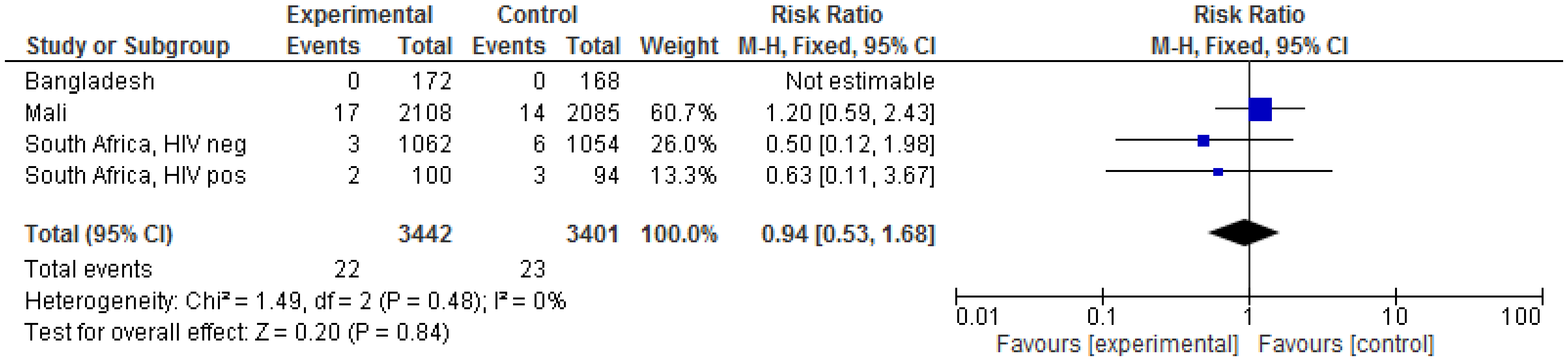

Notes: In Mali, this was reported as infectious death. In South Africa, the details of each death provided in the appendix were reviewed for descriptions indicating an infectious component.

Figure S5. The effect of influenza vaccination in pregnancy on infant non-influenza infectious adverse events within the neonatal period.

	Experimental		Control			Risk Ratio
**Study or Subgroup**	**Events**	**Total**	**Events**	**Total**	**Weight**	**M-H, Fixed, 95% CI**
Bangladesh	0	172	0	168		Not estimable
Mali	60	2108	37	2085	75.5%	1.60 [1.07, 2.41]
South Africa, HIV neg	16	1062	10	1054	20.4%	1.59 [0.72, 3.48]
South Africa, HIV pos	3	100	2	94	4.2%	1.41 [0.24, 8.25]
**Total (95% CI)**		**3442**		**3401**	**100.0%**	**1.59 [1.12, 2.27]**
Total events	79		49			

Heterogeneity: Chi² = 0.02, df = 2 (P = 0.99); I² = 0%
Test for overall effect: Z = 2.59 (P = 0.010)

Risk Ratio
M-H, Fixed, 95% CI

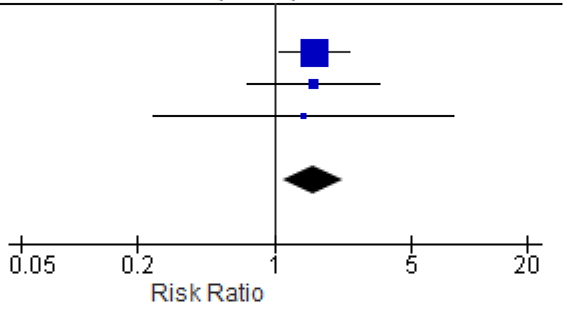

Notes: In the Mali trial, including neonatal infection as reported in the paper. In the South Africa trial, including sepsis<28 days.

References

1. Zaman K, Roy E, Arifeen SE, et al. Effectiveness of maternal influenza immunization in mothers and infants. *N Engl J Med* 2008;359(15):1555-64. doi: 10.1056/NEJMoa0708630 [published Online First: 2008/09/19]
2. Madhi SA, Cutland CL, Kuwanda L, et al. Influenza vaccination of pregnant women and protection of their infants. *N Engl J Med* 2014;371(10):918-31. doi: 10.1056/NEJMoa1401480 [published Online First: 2014/09/04]
3. Tapia MD, Sow SO, Tamboura B, et al. Maternal immunisation with trivalent inactivated influenza vaccine for prevention of influenza in infants in Mali: a prospective, active-controlled, observer-blind, randomised phase 4 trial. *Lancet Infect Dis* 2016;16(9):1026-35. doi: 10.1016/s1473-3099(16)30054-8 [published Online First: 2016/06/05]
4. Steinhoff MC, Katz J, Englund JA, et al. Year-round influenza immunisation during pregnancy in Nepal: a phase 4, randomised, placebo-controlled trial. *Lancet Infect Dis* 2017;17(9):981-89. doi: 10.1016/s1473-3099(17)30252-9 [published Online First: 2017/05/20]
